# Unique trans-kingdom microbiome structural and functional signatures predict cognitive decline in older adults

**DOI:** 10.1007/s11357-023-00799-1

**Published:** 2023-05-22

**Authors:** Diptaraj S. Chaudhari, Shalini Jain, Vinod K. Yata, Sidharth P. Mishra, Ambuj Kumar, Amoy Fraser, Judyta Kociolek, Mariana Dangiolo, Amanda Smith, Adam Golden, Michal M. Masternak, Peter Holland, Marc Agronin, Cynthia White-Williams, Andrea Y. Arikawa, Corinne A. Labyak, Hariom Yadav

**Affiliations:** 1 USF Center for Microbiome Research, Institute for Microbiomes, University of South Florida Morsani College of Medicine, Tampa, FL 33612 USA; 2 Center of Excellence for Aging and Brain Repair, University of South Florida Morsani College of Medicine, Tampa, FL USA; 3 Microbiome in aging Gut and Brain (MiaGB) Consortium Team, FL Tampa, USA; 4 Department of Neurosurgery and Brain Repair, University of South Florida Morsani College of Medicine, Tampa, FL USA; 5 Research Methodology and Biostatistics Core, University of South Florida Morsani College of Medicine, Tampa, FL USA; 6 Burnett School of Biomedical Sciences, University of Central Florida College of Medicine, Orlando, FL USA; 7 Department of Nutrition and Dietetics, University of North Florida, Jacksonville, FL USA; 8 Byrd Alzheimer Center, University of South Florida Morsani College of Medicine, Tampa, FL USA; 9 Department of Neuroscience, Schmidt College of Medicine, Florida Atlantic University, Boca Raton, FL USA; 10 Behavioral Health, MIND Institute, Miami Jewish Health, Miami, FL USA; 11 Present Address: School of Global Health Management and Informatics, University of Central Florida, Orlando, FL USA; 12 Present Address: University of Central Florida College of Medicine, FL Orlando, United States; 13 Department of Head and Neck Surgery, Poznan University of Medical Sciences, Poznan, Poland

**Keywords:** MiaGB, Gut microbiome, Shotgun metagenomics, Cognitive impairment, Aging, Gut-brain axis

## Abstract

The prevalence of age-related cognitive disorders/dementia is increasing, and effective prevention and treatment interventions are lacking due to an incomplete understanding of aging neuropathophysiology. Emerging evidence suggests that abnormalities in gut microbiome are linked with age-related cognitive decline and getting acceptance as one of the pillars of the Geroscience hypothesis. However, the potential clinical importance of gut microbiome abnormalities in predicting the risk of cognitive decline in older adults is unclear. Till now the majority of clinical studies were done using 16S rRNA sequencing which only accounts for analyzing bacterial abundance, while lacking an understanding of other crucial microbial kingdoms, such as viruses, fungi, archaea, and the functional profiling of the microbiome community. Utilizing data and samples of older adults with mild cognitive impairment (MCI; *n* = 23) and cognitively healthy controls (*n* = 25). Our whole-genome metagenomic sequencing revealed that the gut of older adults with MCI harbors a less diverse microbiome with a specific increase in total viruses and a decrease in bacterial abundance compared with controls. The virome, bacteriome, and microbial metabolic signatures were significantly distinct in subjects with MCI versus controls. Selected bacteriome signatures show high predictive potential of cognitive dysfunction than virome signatures while combining virome and metabolic signatures with bacteriome boosts the prediction power. Altogether, the results from our pilot study indicate that trans-kingdom microbiome signatures are significantly distinct in MCI gut compared with controls and may have utility for predicting the risk of developing cognitive decline and dementia- debilitating public health problems in older adults.

**Supplementary Information:**

The online version contains supplementary material available at 10.1007/s11357-023-00799-1.

## Introduction

With an aging world population, cognitive decline and dementia are debilitating public health problems in older adults [17]. Alzheimer’s disease (AD) is the most common age-related cognitive disorder [53]. Around 6 million older adults are living with Alzheimer’s disease and related dementia (ADRD) in the USA, and this number is expected to grow double by 2050 [41]. Currently, there are no clinically impactful prevention strategies and no treatments that can significantly alter the course of the illness. As a result, ADRD causes great strain on families, society, and the healthcare system [12, 39]. Drug clinical trials for treating AD [34, 57] lack a full understanding of ADRD pathophysiology as well as the right targets and time frame to introduce interventions. Prior studies indicate that specific diet and exercise regimens may slow the progression of ADRD in older adults [42, 49]. However, the early detection of cognitive decline and dementia risk is cumbersome, expensive, and not available for routine clinical use [29]. Therefore, development of inexpensive, safe, and easy-to-measure testing is direly needed for slowing or preventing the progression of dementia in older adults.

Emerging evidence suggests that abnormalities in gut microbiome may contribute to aging biology mechanisms [45]. A few studies also indicate that the gut microbiome signatures may be different in older adults with ADRD compared with their age-matched controls [14, 36, 37, 56]. Vogt, et al. showed that *Blautia, Phascolarctobacterium, Gemella, Bacteroides, Bilophila*, and *Alistipes* bacteria (many of them are commensal pathogens) were significantly increased, and *SMB53* (family *Clostridiaceae*), *Dialister*, *Clostridium*, *Turicibacter*, *Bifidobacterium, Adlercreutzia*, and *cc115* (family *Erysipelotrichaceae*) (many of them are beneficial/probiotics) were specifically decreased in gut of AD patients compared to controls [55]. In addition, *Escherichia/Shigella, Ruminococcaceae, Enterococcaceae, and Lactobacillaceae* bacteria were significantly increased and *E. rectale, Lanchnospiraceae*, *Bacteroidaceae*, and *Veillonellaceae* were significantly decreased in older adults with mild cognitive impairment (MCI) and were linked with AD markers in cerebrospinal fluid (CSF) [11, 36, 37, 62].

Gut microbiome signatures are greatly influenced by dietary habits, and impact of dietary manipulations on slowing cognitive decline or dementia progression may be through gut microbiome [2, 15]. We have shown that a modified Mediterranean ketogenic diet (MMKD) may change the gut microbiome composition and ameliorate AD pathology in MCI subjects [36]. However, these studies were aimed at describing the difference in microbiome signatures, but not testing their significance for predicting or differentiating cognitive dysfunctions in older adults. In addition, majority of previous studies have used 16S rRNA sequencing which only allows for analysis of the bacteria population (bacteriome) of gut microbiome. The role and significance of other microbial kingdoms (i.e., viruses, fungi, and archaea) that also coexist with bacteria in the human gut remain unstudied. Herein, we performed whole genome sequencing on fecal DNA samples of older adults (≥ 60 years of age) with MCI and normal cognition from the cohort of the MiaGB (Microbiome in aging Gut and Brain) consortium—a multi-site study focused on examining the relationship between the microbiome and aging [31]. The present study investigated the associations between shotgun metagenomics-based trans-kingdom microbiome signatures and cognitive health by comparing older adults with and without mild cognitive impairment.

## Materials and methods

### Human subjects

The data and samples used in this study were procured from the Microbiome in aging Gut and Brain (MiaGB) Consortium cohort as a pilot study. The MiaGB consortium is recruiting community dwelling older adults in Florida at five sites. All the participants (*n* = 48) included in this study were 60 years of age or older. Among them, 23 were with MCI, while 25 subjects were cognitively healthy controls. Cognitive function assessments were performed as described below. The demographic characteristics are depicted in Table 1. Exclusion criteria consisted of persons with (a) history of brain and gut-related surgeries in the past five years; (b) history of cancer diagnosis and/or treatment (except non-melanoma skin cancer) in the past five years; (c) neurological disorders of epilepsy, Parkinson’s disease, and amyotrophic lateral sclerosis; (d) antibiotic use in the preceding 30 days, (e) diarrhea, vomiting, or food poisoning in the past 30 days; and (f) a history of inflammatory bowel diseases. Informed consent was obtained from each participant. All recruitments, study protocols, and procedures were approved the Institutional Review Board of University of South Florida committee and were performed according to the approved guidelines.

**Table 1:** Demographic information of the study participants

	Controls (*n* = 25)	MCI (*n* = 23)
Male/Female	11/14	6/17
Age	70.7 ± 9	75 ± 10.1
BMI	27.1 ± 4.4	25.9 ± 5.6
Ethnicity		
NOT Hispanic or Latino	25 (100%)	20 (87%)
Hispanic or Latino	-	1 (4%)
Not reported	-	2 (9%)
Race		
White	24 (96%)	17 (74%)
Asian	-	3 (13%)
Black or African American	1 (4%)	1 (4%)
Not reported	-	2 (9%)
MoCA	28.0 ± 1.5	23.1 ± 1.7
MiniCog	4.8 ± 0.5	3.4 ± 1.4
MIS	7.2 ± 0.9	5.9 ± 2.3

### Cognitive function assessments

The Montreal cognitive assessment (MoCA) [23], MiniCog [7], and Memory impairment screen (MIS) [30] were performed by trained staff and scores were calculated using standard protocols.

### Stool sample collection

Fecal microbiome samples were collected using an in-house developed stool sample collection kit, which has been validated and accepted by older adults in several of our past [36, 37] and ongoing clinical studies. The use of this kit has increased the compliance and adherence in our studies. The stool collection kit is given to participants to take home, and samples transported to the lab within 24 h of stool passing and collection, and samples were immediately aliquoted and stored at − 80 °C until further analysis.

### Metagenomic shotgun sequencing

Fecal DNA was extracted using 150 mg of the human stool samples using QIAamp PowerFecal Pro DNA Kit (Qiagen, USA) following the manufacturer’s instructions. The DNA was quantified using Qubit dsDNA HS assay kit (Thermo Fisher Scientific, USA). The extracted and quantified DNA (150 ng) was used for library preparation using Illumina® DNA Prep, (M) Tagmentation kit (Illumina, Inc, 5200 Illumina Way, San Diego CA, USA) by following the manufacturer’s instructions. Additionally, sample specific unique IDT for Illumina–Nextera DNA UD Indexes were used. The sequencing was done on Illumina NextSeq1000 machine using an Illumina NextSeq 1000/2000 P2 Reagents (300 Cycles) v3 reagent cartridge (Illumina, Inc, 5200 Illumina Way, San Diego CA, USA). All the data was captured and stored in the BaseSpace cloud and was analyzed further using bioinformatics pipelines, as described below.

### Bioinformatics and statistical analysis

The analysis for the shotgun sequencing data was performed using the Yet Another Metagenomic Pipeline (YAMP) workflow [54]. The YAMP workflow uses tools from bbmap suite for de-duplication, trimming, and decontamination of metagenomics sequences [10]. It uses FastQC for the visualization of the raw and QC filtered metagenomic reads [1]. The additional tools used in the YAMP pipeline are MetaPhlAn [5] for taxonomic binning and profiling of microbes and their relative abundance in the samples, HUMAnN pipeline for the estimation of the functional capabilities of the microbiome community [5], and QIIME2 [21] for the evaluation of the multiple alpha diversity measures including observed OTUs, Shannon and Simpson alpha diversity. The MetaPhlAn database relies on ~ 1.1 M unique clade-specific marker genes identified from ~ 100,000 reference genomes (~ 99,500 bacterial and archaeal and ~ 500 eukaryotic), which allows unambiguous taxonomic assignments, an accurate estimation of organismal relative abundance, species-level resolution for bacteria, archaea, eukaryotes, and viruses. The HUMAnN pipeline which uses MetaPhlAn and ChocoPhlAn pangenome database to facilitate fast, accurate, and organism-specific functional profiling of Archaea, Bacteria, Eukaryotes, and Viruses considerably expanded databases of genomes, genes, and pathways by mapping the metagenome reads on the reference databases. The β-diversity across the sample groups was estimated using Principal Component Analysis (PCA) based on Euclidean distances. Taxonomic abundance of microbial taxa at phylum and species level are represented. The shared and unique bacterial taxa were estimated using a web-based tool interactiveVenn [22]. Statistical analysis of the data was done using Graphpad Prism [6] and Stamp [40]. Various R-scripts including ggplot2 were used for the analysis and presentation of the data like corrplot for the correlation analysis of microbiome components and the cognitive scores of the study participants. The random forest analysis was performed using the web-based tool microbiome analysts [13].

## Results

### The gut of older adults with MCI harbors significantly distinct transkingdom microbiome signatures than their cognitively healthy counterparts

**Fig. 1:**
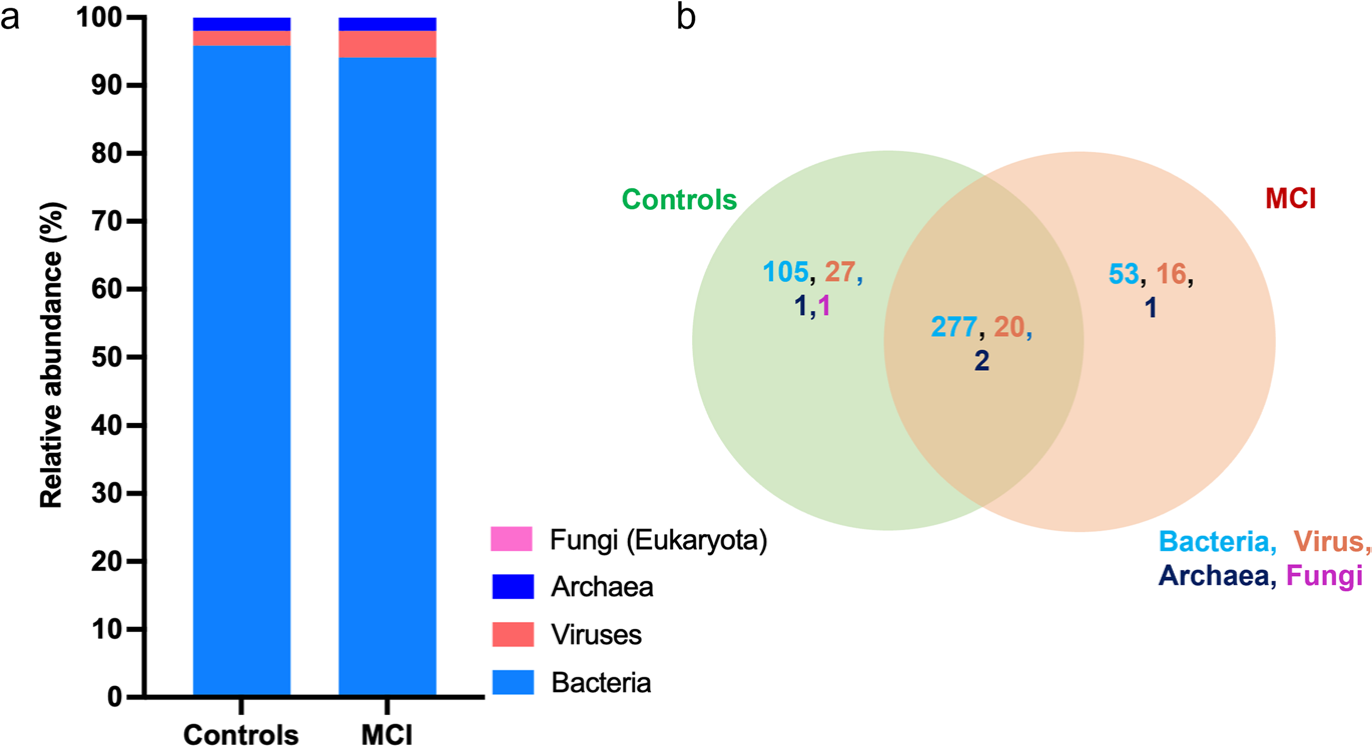
Trans-kingdom microbiome signatures significantly differ in the gut of older adults with mild cognitive impairment (MCI) compared with cognitively healthy controls. **a**) Bar plots depict the mean relative abundance of archaea, bacteria, fungi, and viruses in the gut microbiome of the participants with MCI and controls. **b**) Venn diagram depicting the presence of shared and unique microbial species of the archaea, bacteria, fungi, and viruses in the gut of older adults with MCI and controls

Whole metagenome shotgun sequencing analysis was performed on 48 study participants (23 with MCI and 25 cognitively healthy controls). Taxonomic profiling identified that bacteria comprised the majority of microbiome composition. However, total bacterial abundance was slightly lower in the gut of subjects with MCI than cognitively healthy controls (Fig. 1a). Conversely, the abundance of total viruses was higher in the gut of subjects with MCI than controls (Fig. 1a). An abundance of fungi and archaea were detected in only a few participants. Fifty-three bacteria and 16 viruses were uniquely abundant in the gut of MCI participants and 105 bacteria, and 27 viruses were uniquely present in the gut of controls (Fig. 1b; Supplementary Table S1), suggesting an association between certain gut microbes and differences in cognitive health. The microbiome β-diversity (a measure of microbial diversity between the samples/groups) signatures were not significantly different in gut of MCI and controls (Supplementary Fig. S1a). However, a trend of lower bacterial α-diversity (a measure of microbial diversity within a sample; higher indicates healthier microbiome) was seen in MCI gut compared with controls, while virome α-diversity remained unchanged (Supplementary Fig. S1b-e). These changes in the viral and bacterial α-diversity (both Shannon and Simpson) showed a trend of positive correlation with MoCA scores (cognitive function measure) without achieving statistical significance (Supplementary Fig. S1f-i). Overall, these results indicate that trans-kingdom microbiome (majorly built by bacteria and viruses) were unique and significantly distinct in the gut of older adults according to their cognitive state, and a trend of lower bacterial diversity was linked with poor cognitive function.

### The gut of older adults with MCI harbors distinct virome signature compared with cognitively healthy participants

**Fig. 2:**
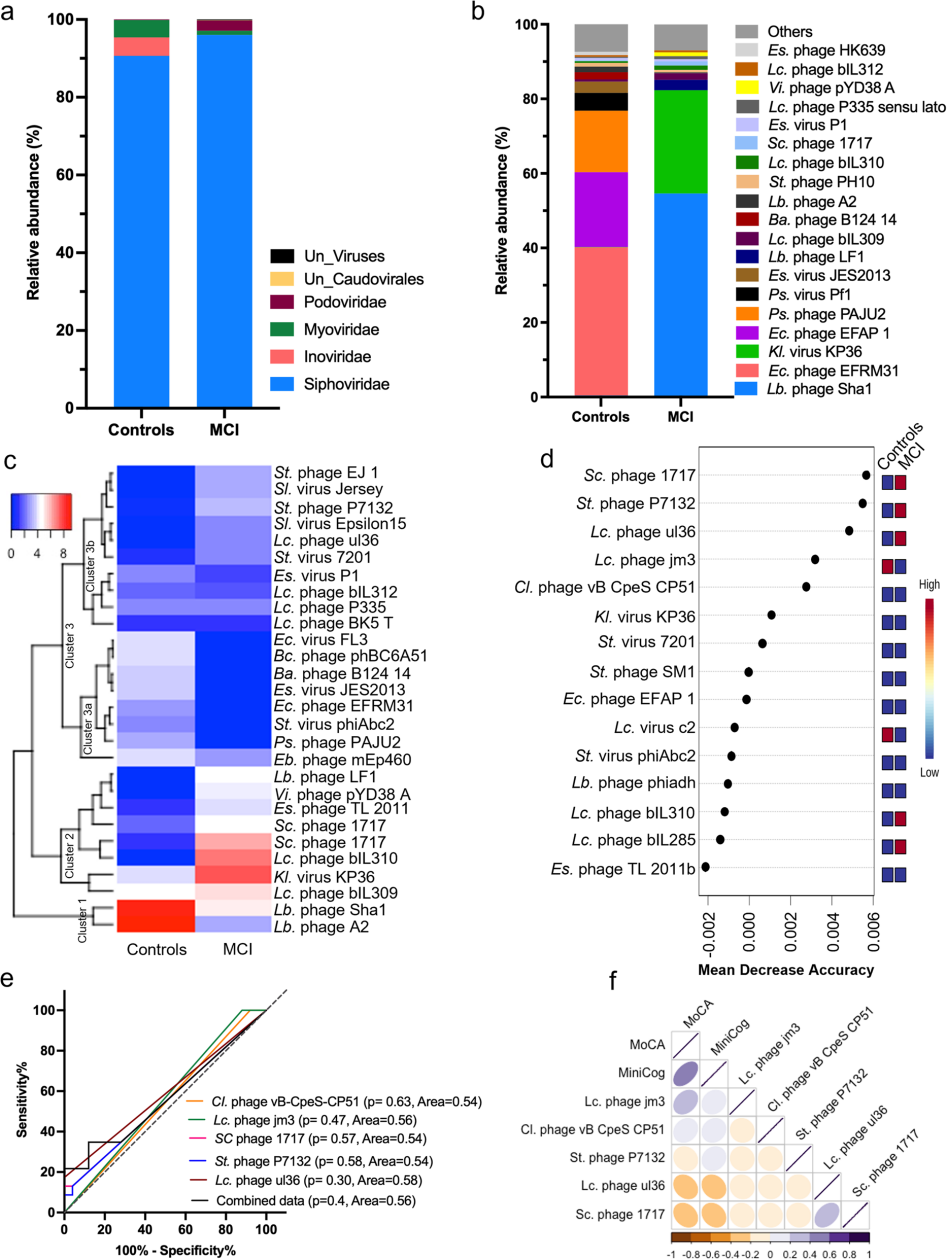
The virome signature is significantly distinct in the gut of older adults with MCI compared with controls, with limited potential to predict cognitive health. **a**,**b**) The relative abundance of major viral families (**a**) and species (**b**) was distinct in the gut microbiome of older adults with MCI compared with controls. **c**) Heatmap depicting the clusters of increased and decreased abundance of viral species in the gut of older adults with MCI and controls. **d**) Random forest analysis (RFA) showing the top 15 viral species with the highest discriminatory power between MCI and control groups. Red color indicates high abundance, and blue indicates a low abundance of the particular viral species in MCI and control groups. (**e**) Receiver operating characteristic (ROC) curve plots represent the specificity and sensitivity of the five selected viral species for the two groups. (**f**) The Pearson correlation matrix shows the association between the relative abundance of selected 5 viral species with cognitive function measures such as MoCA and MiniCog. Abbreviations—*Bacillus; Bc, Bacteroides; Ba, Clostridium; Cl, Enterobacteria; Eb, Enterococcus; Ec, Escherichia; Es, Klebsiella; Kl, Lactobacillus; Lb, Lactococcus; Lc, Pseudomonas; Ps, Salmonella; Sl, Streptococcus; St, Stx2 converting; Sc, Vibrio; Vi*

A virome signature comprising *Podoviridae*, *Inoviridae*, *Myoviridae*, and *Siphoviridae* viral families was distinctly abundant in the gut of older adults with MCI and cognitively healthy controls (Fig. 2a); however, the clustering of virome signatures in principal component analysis (PCA) was not distinct in MCI versus controls (Supplementary Fig. S2). *Lactobacillus (Lb.)* phage Sha1*, Klebsiella (Kl.)* virus KP36*, Lb.* phage LF1, and *Lactococcus (Lc.)* phage bIL309 were uniquely and significantly increased in the gut of MCI, while *Enterococcus (Ec*.) phage EFRM31*, Ec.* phage EFAP 1*, Pseudomonas (Ps.)* phage PAJU2*, Ps.* phage Pf1*, Escherichia (Es*.) virus JES2013*,* and *Bacteroides (Ba.)* phage B124 14 were uniquely increased in the gut of cognitively healthy controls (Fig. 2b). Heatmap and dendrogram of hierarchical clustering analysis revealed three significantly distinct clusters of viral species such as cluster 1 (containing *Lb.* phage A2 and *Lb.* phage Sha1) and cluster 2 (containing *Lc.* phage bIL309*, Kl.* virus KP36*, Lc.* phage bIL310*, Stx2 converting (Sc.)* phage 1717*, Es.* phage TL 2011*, Vibrio (Vi.)* phage pYD38 A, *and Lb.* phage LF1) were increased in the gut of MCI compared to the controls (Fig. 2c). Similarly, within cluster 3, two clear subclusters were apparent, in which cluster 3a (containing *Enterobacteria (Eb.)* phage mEp460, *Ps.* phage PAJU2, *Streptococcus (St.)* virus phiAbc2, *Ec.* phage EFRM31, *Es.* virus JES2013, *Ba.* phage B124 14, *Bacillus* (*Bc*.) phage phBC6A51 and *Ec.* virus FL3) was reduced, while cluster 3b (containing *Streptococcus (St.)* phage EJ 1, *Salmonella (Sl.)* virus Jersey, *St.* phage P7132, *Sl.* virus Epsilon15, *Lc.* phage ul36 and *St.* virus 7201) was increased in the gut of MCI subjects compared to their controls (Fig. 2c). Further, random forest analyses to determine the unique signature of virome with predictive potential showed that the *Sc.* phage 1717*, St.* phage P7132*, Lc.* phage ul36*, Lc.* phage jm3*, Clostridium (Cl.)* phage vB CpeS CP51*, Kl.* virus KP36*, St.* virus 7201*, St.* phage SM1*, Ec.* phage EFAP 1*, Lc.* virus c2*, St.* virus phiAbc2*, Lb.* phage phiadh*, Lc.* phage bIL310*, Lc.* phage bIL285, and *Es.* phage TL 2011b were significantly distinct between MCI and controls with potential to be used as biomarkers (Fig. 2d)*.* To further, test their ability to diagnose cognitive decline, a Receiver Operating Characteristic (ROC) curve (a graphical plot used to show the diagnostic ability of binary classifiers) method show that the abundance of *Cl.* phage vB CpeS CP51*, Lc.* phage jm3*, Sc.* phage 1717*, St.* phage P7132*, and Lc.* phage ul36 show an area under curve (AUC) of 0.54, 0.54, 0.58, and 0.56 to 0.58 (Fig. 2e), suggesting that these individual viral species have 54 to 58% confidence/ability to discriminate MCI from cognitively healthy controls. In addition, the combination of these five viral species showed 0.56 ROC suggesting that altogether these viral species have a limited diagnostic potential individually as well as their combination together. Pearson correlation analyses also indicated that the selected viral species were significantly correlated with cognitive function measures MoCA and MiniCog scores, though, *Lc.* phage ul36 and *Sc.* phage 1717 showed highest correlation with MoCA (*r*^2^ = 0.06 and *r*^2^ = 0.07 respectively) and MiniCog (*r*^2^ = 0.04 and *r*^2^ = 0.05, respectively) scores (Fig. 2f), suggesting that the increased abundance of these viral species may be indicators of cognitive decline in older adults.

### The bacteriome signature in gut of older adults with MCI was significantly distinct than in cognitively healthy controls with potential to differentiate them

**Fig. 3:**
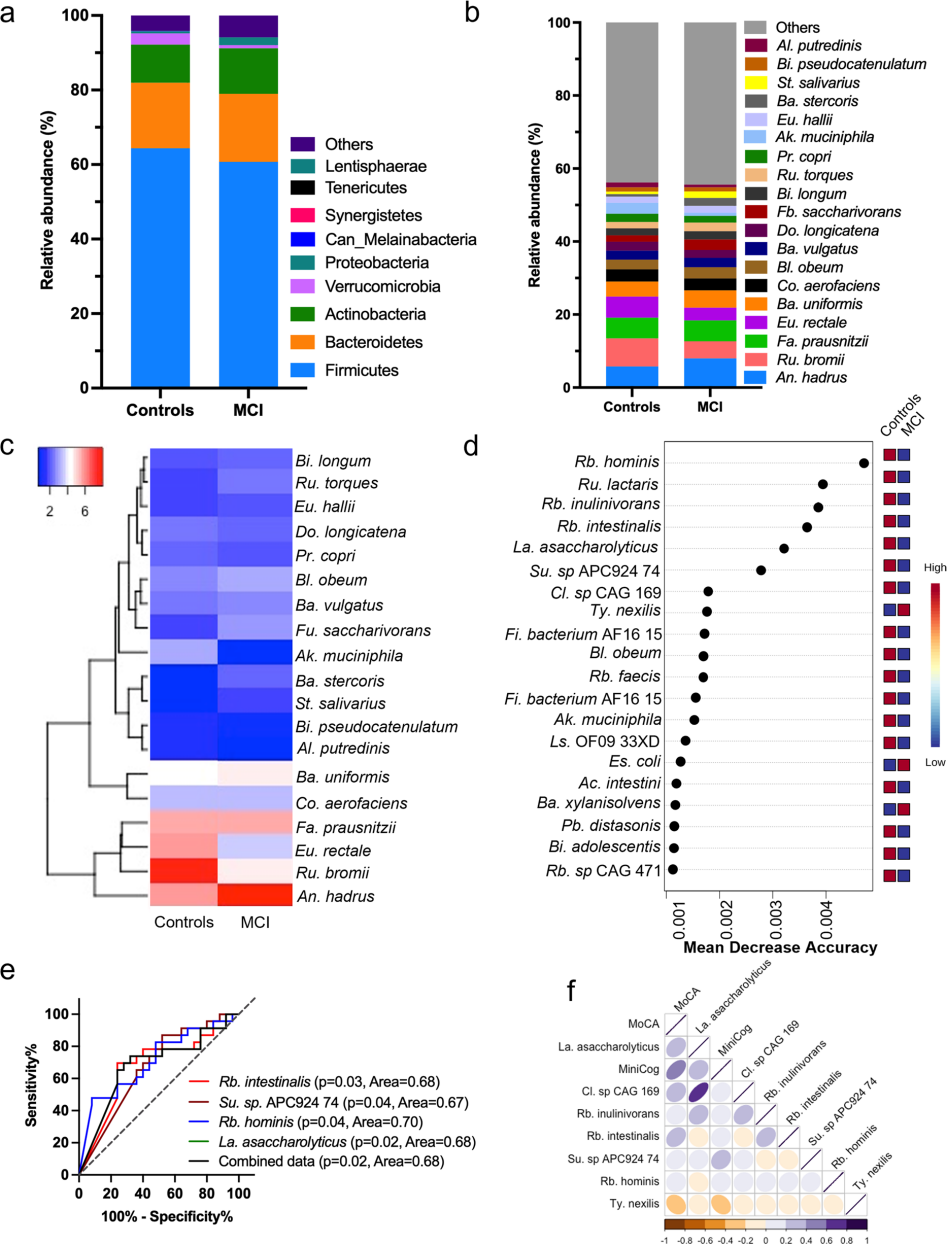
The bacteriome signatures in the gut of older adults with MCI significantly differ from cognitively healthy controls with a moderate predictive potential of cognitive health. **a**,**b**) The relative abundance of major bacterial phyla (**b**) and species (**b**) in the gut of the older adults with MCI in comparison to controls. (**c**) Heatmap depicting the group-specific enrichment of the bacterial species in the gut of older adults with MCI and controls. (**d**) Random forest analysis showing the top 15 bacterial species with the highest discriminatory power between the control and MCI groups. (**e**) ROC analyses of selected bacterial species to predict the cognitive health in older adults. (**f**) Correlation matrix showing the association between the relative abundance of selected bacterial species with MoCA and Mini-Cog. Abbreviations*—Acidaminococcus; Ac, Akkermansia; Ak, Alistipes; Al, Anaerostipes; An, Bacteroides; Ba, Bifidobacterium; Bi, Blautia; Bl, Clostridium; Cl, Collinsella; Co, Dorea; Do, Escherichia; Es, Eubacterium; Eu, Faecalibacterium; Fa, Firmicutes; Fi, Fusicatenibacter; Fb, Fusicatenibacter; Fu, Lachnospiraceae; Ls, Lawsonibacter; La, Parabacteroides; Pb, Prevotella; Pr, Roseburia; Rb, Ruminococcus; Ru, Streptococcus; St, Subdoligranulum; Su, Tyzzerella; Ty*

The abundance of major phyla was distinct in gut of MCI than their controls (Fig. 3a), without significant differences in the microbiome diversity indices (both α- and β-diversity) between these groups (Supplementary Fig. S1a, b-e; S3a). The abundance of Bacteroidetes, Actinobacteria, and Proteobacteria was higher, while Firmicutes, Verrucomicrobia, and Synergistetes were lower in the gut of MCI than controls (Fig. 3a), but these differences were marginal or non-statistically significant. Among major bacterial species *An. hadrus* and *Bl. obeum increased while Ru. bromii and Eu. rectale* decreased in the gut of MCI than controls (Fig. 3b). Similarly, hierarchical clustering indicates that the abundance of *Ru. torques, Eu. hallii, Ba. stercoris, St. salivarius*, and *An. hadrus* was higher while *Bi. pseudocatenulatum, Al. putredinis,* and *Ak. muciniphila* were lower in gut of MCI than controls (Fig. 3c). The differential abundance analysis shows that *Ruminococcus (Ru.) lactaris, Su. sp.* APC924*, Eu. siraeum, La. asaccharolyticus, Sl. isoflavoniconvertens, Fi. bacterium* CAG 137*, Cl. sp* CAG 273*, Gemmiger (Ge.) formicilis*, and *Rb. intestinalis* reduced, while *Bl. wexlerae, Bi. Bifidum, Ba. stercoris, Catabacter (Ca.) hongkongensis, Eu. eligens, Cl. bolteae,* and *Phascolarctobacterium (Pb.) faecium* increased in the gut of MCI compared to controls (Supplementary Fig. 3b-g). Further, random forest analysis revealed that *Rb. hominis*, *Ru. lactaris, Rb. inulinivorans, Rb. intestinalis, La. asaccharolyticus, Su. sp* APC924 74*, Ty. nexilis, Es. coli*, and *Ba. xylanisolvens* were significantly distinct between MCI and control with potential to be used as predictive markers for differentiating MCI from cognitively healthy controls (Fig. 3d). The LEfSe (linear discriminant analysis effect size) analysis commonly used for the high dimensional data biomarker discovery also observed the majority of microbiome signatures including the higher abundance of bacterial species *Rb. hominis, Rb. intestinalis, Su. sp.* APC924 in the control group and identified as the important biomarkers associated with the cognitive state of the study participants (Supplementary Fig. 4a, b), like random forest analyses. The ROC analysis shows that the four selected single bacterial species (*Rb. intestinalis, Su. sp* APC924 74*, Rb. hominis*, and *La. asaccharolyticus)* have each around 67–70% power to differentiate the MCI from controls (*p* > 0.05) (Fig. 3e). Interestingly, the combination of these four selected bacterial species showed similar power to differentiate MCI from controls (0.68 AUC or 68% confidence). These four bacterial species showed significant association with MOCA and MiniCog (Fig. 3f). Together, these results indicate that the bacteriome signatures are significantly distinct in MCI compared to controls and have moderate power to differentiate the cognitive health in older adults.

### Metabolic functions of gut microbiome in the gut of older adults with MCI significantly differs from controls

**Fig. 4:**
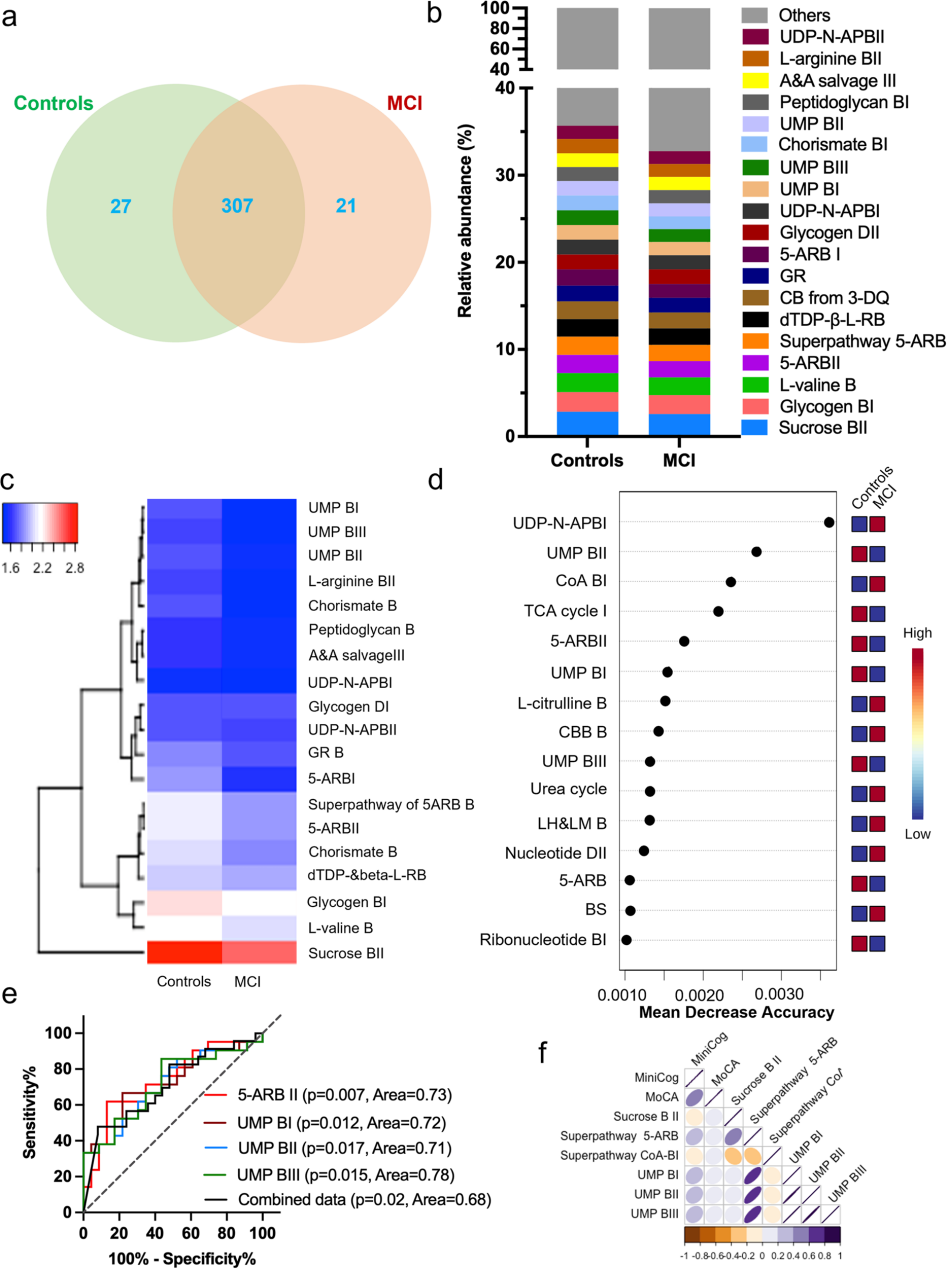
The functional metabolic pathways of the microbiome in the gut of older adults with MCI were significantly distinct from their controls with a moderate predictive potential of cognition. **a**) Venn diagram representing the shared and unique microbial metabolic pathways in the gut of older adults with MCI compared to their controls. **b**) The relative abundance of the top 20 microbial metabolic pathways that are distinct between older adults with MCI and controls. **c**) Heatmap representing the group-specific enrichment of the pathways in the control and MCI participants. **d**) Random forest analysis showing the top 15 pathways with the highest discriminatory power between the control and MCI groups (**e**) ROC analysis showing the specificity and sensitivity of the four selected pathways with discriminating potential between MCI anda controls (**f**) Correlation matrix showing the association of the relative abundance of microbiome functional pathways with MoCA and Mini-Cog. Abbreviations*—*5-Aminoimidazole Ribonucleotide Biosynthesis I; 5-ARB, Acetylmuramoyl-pentapeptide; AP, Adenine and Adenosine; A&A, Biosynthesis; B, Building Blocks Biosynthesis; BB B, Corismate biosynthesis from 3-dehydroquinate; CB from 3DQ, Coenzyme A; CoA, Degradation; Guanosine ribonucleotides; GR, L-homoserine and L-methionine; LH and LM, Rhamnose; R, Tricaboxylic Cycle; TCA, Uridine 5'-monophosphate; UMP

The HMP Unified Metabolic Analysis Network (HUMAnN 3.0) [5] analyses depicted that the metabolic functions of the microbiome were significantly distinct in the gut of older adults with MCI compared to their controls (Fig. 4). PCA analysis plot of microbial metabolic pathways of the gut microbiome of older adults with MCI and controls showed marginal clustering across the axis 2 (Supplementary Fig. S5). A total of 355 pathways were detected, in which 27 were exclusively present in controls and 21 were uniquely detected in the MCI participants (Fig. 4a, Supplementary Table S2). The abundance of 5-Aminoimidazole Ribonucleotide Biosynthesis (5-ARB) namely 5-ARBI and 5-ARBII, as well as pathways relating to the synthesis of the Uridine MonoPhosphate (UMP) viz. UMP BI, UMP BII, and UMP BIII, was lower in the gut microbiome of MCI than controls (Fig. 4b). These observations were further confirmed in the heatmap of the hierarchical clustering showing that all the major microbial metabolic pathways reduced in MCI compared to controls (Fig. 4c). In addition, the differential analysis revealed that 45 pathways were differentially abundant between the MCI and control groups (Supplementary Table S3). Among these 11 pathways including the superpathway of 5-ARB and 5-ARBII, Chorismate biosynthesis (CB) from 3-dehydroquinate, 5-ARBI, CBI, L-histidine biosynthesis, superpathway of L-tyrosine biosynthesis, mannan degradation, 2-oxobutanoate degradation I, formaldehyde assimilation III (dihydroxyacetone cycle), and isopropanol biosynthesis were downregulated in MCI participants compared to controls. While 34 pathways including peptidoglycan maturation, guanosine nucleotides degradation III, seleno-amino acid biosynthesis, adenosine nucleotides degradation II, phosphatidylglycerol biosynthesis I, L-methionine biosynthesis II, purine nucleotides degradation II, guanosine nucleotides degradation II, fatty acid elongation, oleate biosynthesis IV (anaerobic), and (5Z)-dodecenoate biosynthesis I were upregulated in the gut of MCI than controls (Supplementary Table S3). Further, random forest analyses revealed that the upregulated UDP-N-acetylmuramoyl-pentapeptide biosynthesis I (UDP-N-APBI) and Co-A BI and downregulated UMP BII, TCA cycle, and 5-ARBII were among the top 5 pathways detected as the most significant to be useful for prediction of cognitive function in the MCI and control groups (Fig. 4d). The ROC analyses performed on all the pathways selected on random forest analyses demonstrated that the individual pathways like 5-ARBII, UMPBI, UMPBII, and UMPBIII showed statistically significant area under the curve, suggesting predictive power of 71–78% confidence. The combination of these pathways showed 68% predictive power (Fig. 4e). In addition, the increased abundance of UMP BI, UMP BII, and UMP BIII was positively correlated with MoCA and MiniCog scores, while UDP-N-APBI and superpathway of CoA BI were negatively correlated with these cognitive function markers (Fig. 4f). Altogether, these results suggest that the metabolic pathways of microbiome are significantly distinct in the gut of older adults with MCI compared with controls, and these differences can moderately predict cognitive state in older adults.

### Combining virome, bacteriome, and microbial metabolic signatures boosts the prediction of cognitive impairment in older adults

**Fig. 5:**
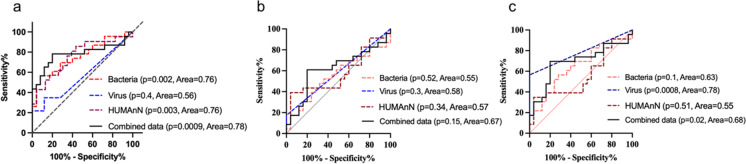
The combination of bacteriome, virome, and microbial metabolic pathways improves the prediction of cognitive health in older adults. **a**–**c**) ROC analyses depicting prediction model 1 (**a**), 2 (**b**), and 3 (**c**) with a distinct combination of bacteriome, virome, and microbial metabolic pathways to predict the cognitive health of older adults

Our findings indicate that the gut of older adults with MCI harbors a significantly distinct trans-kingdom microbiome (virome, bacteria, fungi, and archaea), which show moderate predictive strength. *Rb. hominis* was detected as the top contributor among the bacteria, virus taxa, and metabolic pathways (Supplementary Fig. S5) One limitation, however, is the fact that the viral and bacterial species and metabolic pathways shortlisted in the above analyses are not detected in the microbiome signature of all the participants. To make our results widely applicable, using multi-omics approach, we tested the combination of virome, bacterial, and microbial metabolic signatures that were detected in all the samples. We performed three model combinations using ROC analyses. *Model 1* included bacterial and viral species along with microbial metabolic pathways that were significantly different using random forest analyses (Fig. 5a). This model showed an area under curve of 0.78 (78% confidence) in comparison to 0.76 by bacteria alone, 0.56 by viruses alone and 0.76 by metabolic pathways alone indicating that combining selected bacteriome, virome, and metabolic pathways slightly boosts the predictive power for differentiating MCI from controls. *Model 2* included bacterial, viral, and microbial metabolic pathways that were distinct between MCI and control participants. The prediction power was significantly decreased (0.67 AUC), however boosted within the differentially abundant distinct kingdom signatures. In addition, we also tested if combining model 1 and 2 with uniquely abundant taxa in MCI (*model 3*) can boost the prediction, but we did not see improvements in predictive power (Fig. 5b). However, unique viruses showed highest predictive potential (0.78 AUC) in model 3 (Fig. 5c). These results indicate that the multi-omics analyses of bacteriome, virome, and functional analyses boost the predictive potential to detect cognitive impairment in two ways—(1) increasing its coverage in all the samples and (2) boosting the prediction. Therefore, selected microbiome signatures can be used for developing markers to boost the risk of cognitive decline in older adults.

## Discussion

Gut microbiome composition and functions are known to be significantly different between healthy older adults and young adults as well as between older adults with cognitive disorders like AD and cognitively healthy adults [8, 9, 14, 36, 37, 47, 56]. Similar observations are noted in animal models [26, 60]. However, the results are inconsistent and largely based on targeted 16S rRNA sequencing, which accounts only for bacterial abundance. It is evident that the microbiota is a complex community comprising bacteria, viruses, fungi, and archaea, which together, are interconnected to survive and function. However, the relationship of these microbiome communities in aging biology and cognitive health is poorly understood. Earlier studies show that the gut bacterial population (bacteriome) is variable, which limits its potential to be used as a biomarker to predict cognitive decline in older adults. Furthermore, it is unclear if combining bacteria, viruses, and their metabolic pathways using combinatorial approach can be used to predict cognitive decline. Herein, our results show that the gut of older adults with MCI harbors not only distinct bacteria, but also distinct viruses, with limited number of fungi and archaea detected, and we show that combining the bacteriome, virome, and their metabolic pathways boosts the predictive potential of microbiome to differentiate MCI from cognitively healthy older adults.

The microbiome α-diversity is an indicator of function of the microbiota (higher is better), and we show that the gut of older adults with MCI has lower microbiome α-diversity indices like Shannon and Simpson indexes, which were positively associated with reduced MoCA and MiniCog scores (lower scores indicate poor cognitive function). These results indicate that the gut of older adults with MCI harbors a significantly distinct microbiome compared with the gut of cognitively healthy older adults. Multiple emerging studies indicate associations between gut microbiota diversity and taxonomic signatures with neurological outcomes, including cognitive function and dementia [29, 42]. Preclinical studies using germ-free or antibiotic-treated rodents show no or reduced microbiome diversity, respectively with significant cognitive deficits such as reduced memory, impaired working memory, and changes in brain-derived neurotrophic factor in the hippocampus [19, 32, 35, 43]. Small-scale human studies including ours also showed a link between abnormalities in microbial features and cognition dysfunctions, or found significant improvements when comparing controls with persons who have been treated with probiotics or Mediterranean ketogenic diet to increase commensal microbiota [43]. Our findings are consistent with results from animal models and other clinical studies and advance the understanding of trans-kingdom differences of bacteria and viruses corresponding to the cognitive function in the gut of older adults.

The mechanisms by which the gut microbiome is associated with cognitive health are not fully established yet; however, growing data indicate that microbiota-produced beneficial metabolites such as short-chain fatty acids (SCFAs like acetate, propionate, and butyrate) significantly contribute in gut-brain communications [16, 24]. Herein, we observed that the abundance of butyrate producing bacteria such as *Lachnospiraceae* family, *Subdoligranulum sp., Roseburia intestinalis,* and *Roseburia hominis* were reduced in the gut of MCI participants compared to cognitively healthy controls. It has been previously reported that a decline in abundance of butyrate producing bacteria is associated with multiple disorders such inflammatory bowel disease, type 2 diabetes mellitus as well as poor intestinal barrier function, immune dysregulation, and gut dysbiosis [4, 38, 51, 52, 59]. In the mice study, the increased abundance of *Lachnospiraceae* family was associated with reduced deposition of β amyloid in brain tissue [35]. Butyrate administration to animals has shown protective effects against vascular dementia, cognitive impairment, and against metabolic risk factors for cognitive decline and dementia [3, 20, 27, 28, 46]. Previous studies performed in our team also demonstrated that feeding modified Mediterranean-ketogenic diet (MMKD) increased production of butyrate, which was associated with reduced AD markers in the cerebrospinal fluid of older adults with MCI [36]. These studies suggest that the reduced abundance of butyrate producing bacteria is associated with higher risk of cognitive decline, and butyrate supplementation shows protective effects against cognitive decline in animal models. It remains to be determined the role of bacterial abundance in prediction of cognitive decline risk and whether butyrate therapies can be effective to prevent and/or treat cognitive decline in human populations. Therefore, further studies using larger human cohorts are needed to confirm these findings and test translation of findings from animal models to humans.

Although, bacteria and their metabolites play an important role in regulating gut-brain axis function and cognitive health, evidence also shows that cognitive impairment is associated with viral infections either through direct invasion to the central nervous system or through an indirect effect by inducing systemic inflammation, cytokine storm, hypercoagulability, and neuro-inflammation [48]. In this study, we show that the overall abundance of viruses increased in the gut of older adults with MCI compared with controls. We also observed an abundance of *Podovirideae, Inovirideae, Myovirideae*, and *Siphovirideae*, which belong to bacteriophage types of viral families [18, 25, 58] detected in the human gut and are associated with cognitive function. Recently [33] et al. showed that the higher abundance of *Lactobacillus* phages (family *Siphoviridae* of the order Caudovirales positively associated with better cognitive function; and transplantation of these phages from humans to mice and Drosophila showed increased memory scores and upregulation of memory-involved brain genes. Bacteriophages influence the bacterial composition by impacting their survival and functions, thus contributing to shaping the microbiome diversity, structure, and function [50]. We showed that older adults with MCI had higher abundance of *Lactococcus phage ul36* which specifically regulates the probiotic bacteria like *Lactococcus lactis* (a common yogurt culture) [44] and may diminish their abundance in the gut, which may ultimately be detrimental for gut-brain axis. On other hand, certain bacteriophages exhibit prophage-like properties, such as *Stx2 converting* phage 1717, which functions as a mobile genetic element in bacterial genome containing crucial genes associated with bacterial pathogenesis [61]. The virome signatures detected in the present study were highly variable, and their precise role in age-related cognitive decline remains to be determined.

The importance of microbiome signature differences is debatable due to the high variability in their diversity, taxonomic features, and functions. In this study, we tested the potential of bacteriome, virome, and their metabolic signatures to predict the cognitive health in older adults. Interestingly, we found that the virome showed the highest number of unique viral species in the gut of MCI versus cognitively healthy controls and the strongest predictive power. However, one limitation of using the virome is that viruses are not uniformly present in the human gut. Bacterial species chosen based on differential microbiome signatures and random forest analyses showed significant potential to differentiate (~ 76%) MCI from their cognitively healthy controls. We also observed that the one bacterial species representing a specific enterotype presents more predictive power to differentiate MCI from controls; however, single species or that particular enterotype was not present in all the samples, instead of present in limited samples. Thus, our approach of using combination of virome, bacteriome, and metabolic signatures presented broader application for all the individuals. Interestingly, the addition of selected signatures of virome and microbial metabolic pathways to bacteriome signatures boosted the power to predict the risk of cognitive decline in older adults.

Our study presents several strengths: our results are derived from community dwelling older adults rather than institutionalized patients; the microbiome sequencing was done using whole genome metagenomics which allowed us to identify bacteria, viruses, fungi, and archaea altogether, and we used the well-established cohort of MiaGB consortium which uses standardized protocols for all data collection and quality control, including participant surveys, cognitive functions assessments, stool collection, processing, and sequencing.

We acknowledge that our study also has few limitations. Our sample size is relatively small for comprehensive analysis of multiple microbial signatures, and we were also not able to determine the potential role of sex, race, and ethnicity. We attempted to understand the association of cognitive health and microbiome of the study participants. However, no statistically significant differences were recorded in this cross-sectional study. Nonetheless, the present study was an exploratory analysis of initial data collected in the MiaGB consortium, which is a cohort study aimed at recruiting approximately 400 older adults and following them on a yearly basis. Further analyses to use and validate the findings of this study will be performed in the future. One immediate use will be to use these results as a training cohort/dataset to test the efficacy and accuracy of these predictions. Lastly, while the current study is cross-sectional, which prevents the assessment of temporality in microbiome signatures as longitudinal data from participants in the MiaGB consortium become available, we will be able to define the temporal changes associated with trans-kingdom signatures so we can better understand the relationship between these and cognitive health.

### Supplementary Information

Below is the link to the electronic supplementary material.

Table S1 (XLSX 20 kb)

Table S2 (XLSX 16 kb)

Table S3 (XLSX 11 kb)

Table S4 (XLSX 17 kb)

Supplementary Fig. S1 Trans-kingdom microbiome diversity and their link with cognitive function measures in older adults. a) Microbiome signature in terms of β-diversity at the trans-kingdom level. b-e) The α-diversity indices Shannon (b,d) and Simpson (c,e) of virome (b,c) and bacteriome (d,e) in the gut of older adults with MCI and cognitively healthy controls. f-i) The spearman correlations of Shannon (f,h) and Simpson (g,i) diversity indices of virome (f,g) and bacteriome (h,i) with cognitive function measured by MoCA. (PNG 1492 kb)

High resolution image (TIF 871 kb)

Supplementary Fig. S2 PCA plot depicting the virome microbiome signature in the gut of older adults with MCI and controls. (PNG 220 kb)

High resolution image (TIF 636 kb)

Supplementary Fig. S3 a) PCA plot depicting the β-diversity of bacteriome signature. b) Volcano plot showing the differentially abundant bacterial species in the gut of older adults with MCI compared with the controls. c-g) Box plot depicting the relative abundance of *Subdoligranulum (Su)* sp. APC924-74 (c), *Roseburia (Rb.) hominis* (d), *Lawsonibacter (La.) asaccharolyticus* (e), *Roseburia (Rb.) intestinalis* (f) and *Eubacterium (Eu.) siraeum* (g) in the gut of older adults with MCI and controls. *P* values ≤ 0.05 are statistically significant (*t*-test). (PNG 1001 kb)

High resolution image (TIF 1958 kb)

Supplementary Fig. S4 a) Cladogram indicating the phylogenetic distribution of the bacterial lineages associated with the control and MCI groups. Circles indicate phylogenetic levels. The diameter of each circle is proportional to the abundance of the given bacterial taxon. b) Linear discriminant analysis Effect Size (LEfSe) analysis on microbiome taxa among the control and MCI groups. (PNG 1544 kb)

High resolution image (TIF 2492 kb)

Supplementary Fig. S5 PCA analysis plot of microbial metabolic pathways of the gut microbiome of older adults with MCI and controls. (PNG 323 kb)

High resolution image (TIF 874 kb)

Supplementary Fig. S6. Random forest analysis (RFA) depicting the top 15 contributors, including bacteria, pathways, and viruses with the highest discriminatory power between the control and MCI groups. (PNG 348 kb)

High resolution image (TIF 605 kb)

## Data Availability

The metagenomic sequencing data is available at NCBI SRA bio-project no PRJNA912638.
