# Supplementary figures and images for: Unique trans-kingdom microbiome structural and functional signatures predict cognitive decline in older adults
Source: GeroScience. 2023 May 22;45(5):2819–34. doi: 10.1007/s11357-023-00799-1 (PMC10643725; doi:10.1007/s11357-023-00799-1)

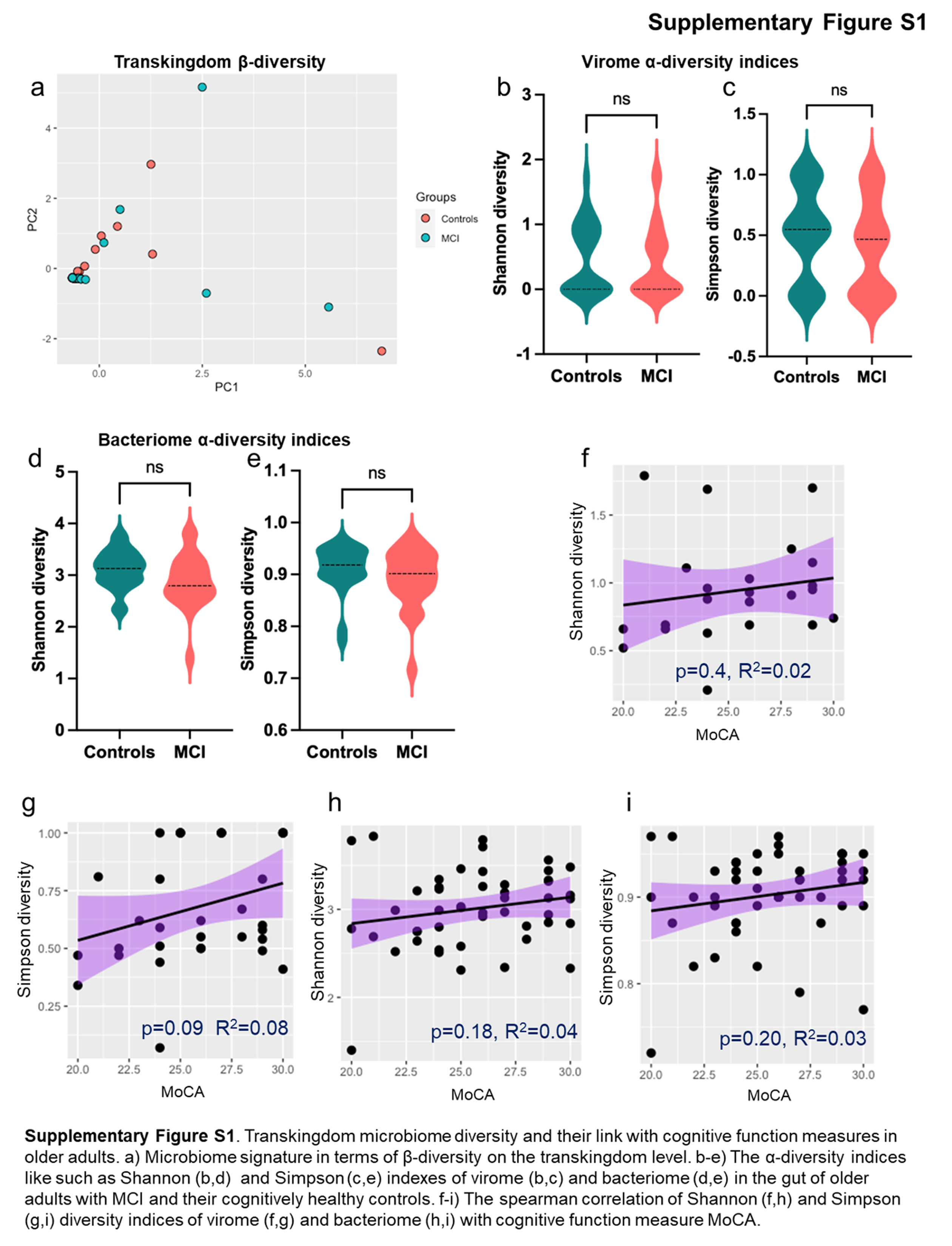

Supplement: Supplementary file 5 — Trans-kingdom microbiome diversity and their link with cognitive function measures in older adults. a) Microbiome signature in terms of β-diversity at the trans-kingdom level. b-e) The α-diversity indices Shannon (b,d) and Simpson (c,e) of virome (b,c) and bacteriome (d,e) in the gut of older adults with MCI and cognitively healthy controls. f-i) The spearman correlations of Shannon (f,h) and Simpson (g,i) diversity indices of virome (f,g) and bacteriome (h,i) with cognitive function measured by MoCA. (PNG 1492 kb) [file 11357_2023_799_Fig6_ESM.png]

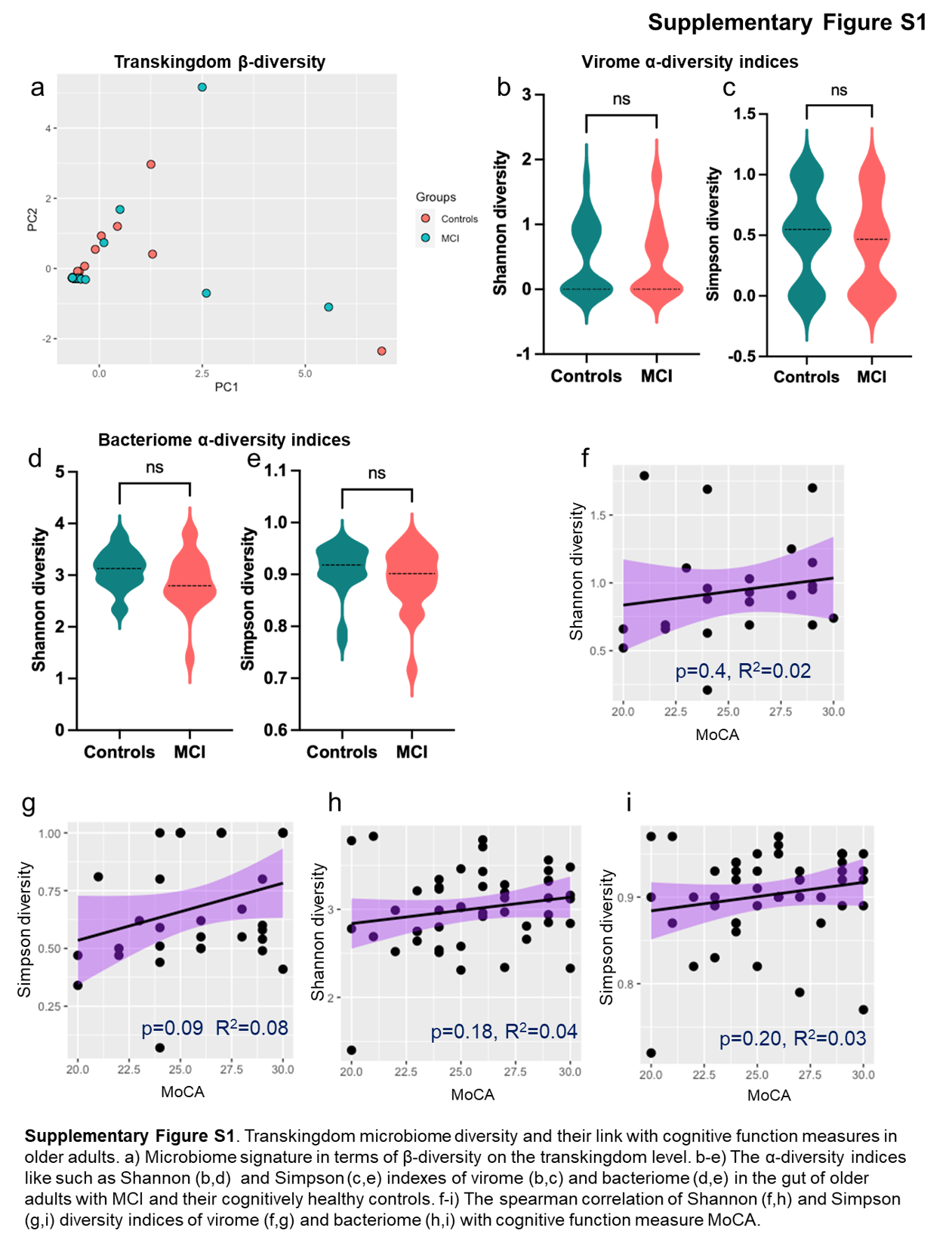

Supplement: Supplementary file 6 — High resolution image (TIF 871 kb) [file 11357_2023_799_MOESM5_ESM.tif]

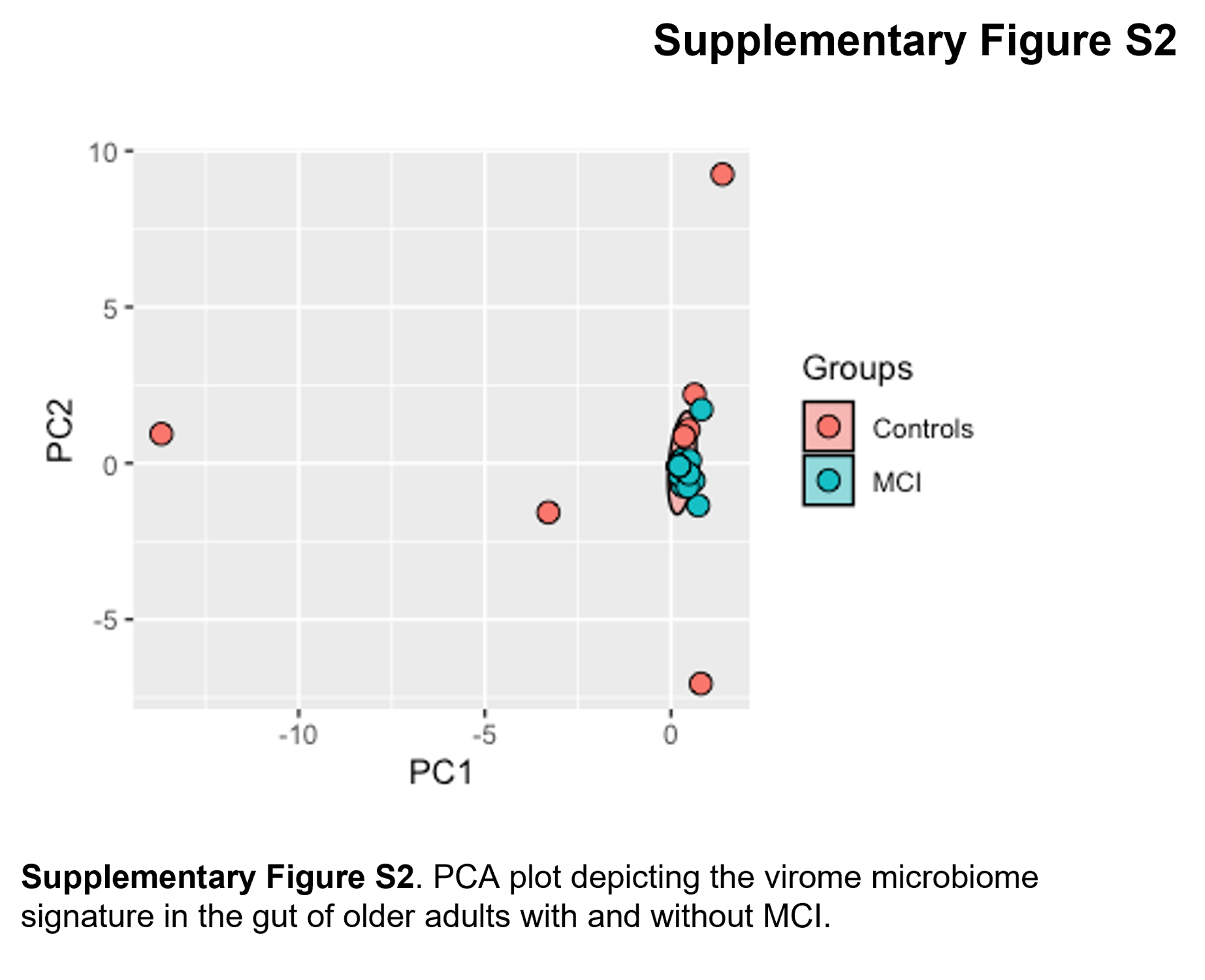

Supplement: Supplementary file 7 — PCA plot depicting the virome microbiome signature in the gut of older adults with MCI and controls. (PNG 220 kb) [file 11357_2023_799_Fig7_ESM.png]

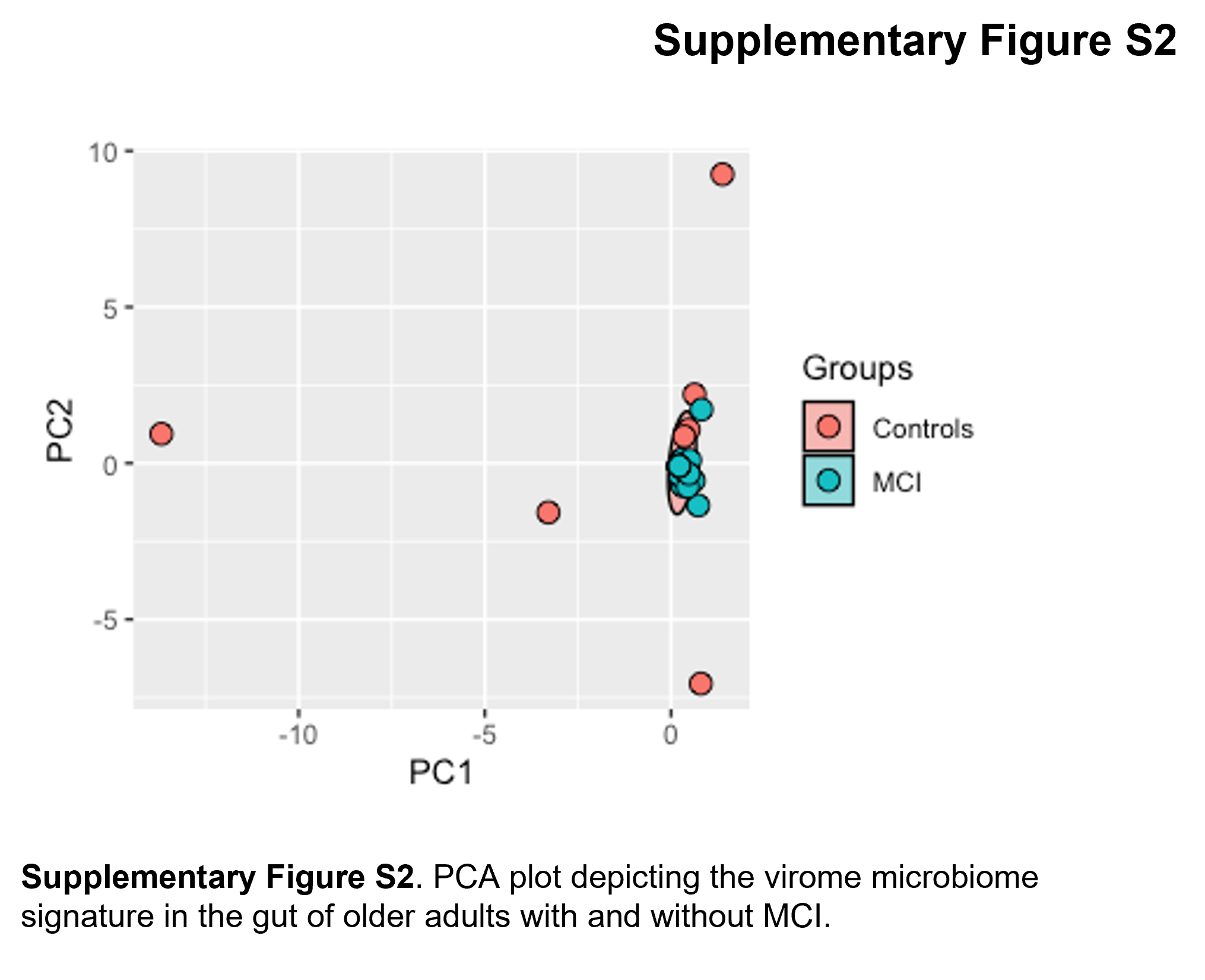

Supplement: Supplementary file 8 — High resolution image (TIF 636 kb) [file 11357_2023_799_MOESM6_ESM.tif]

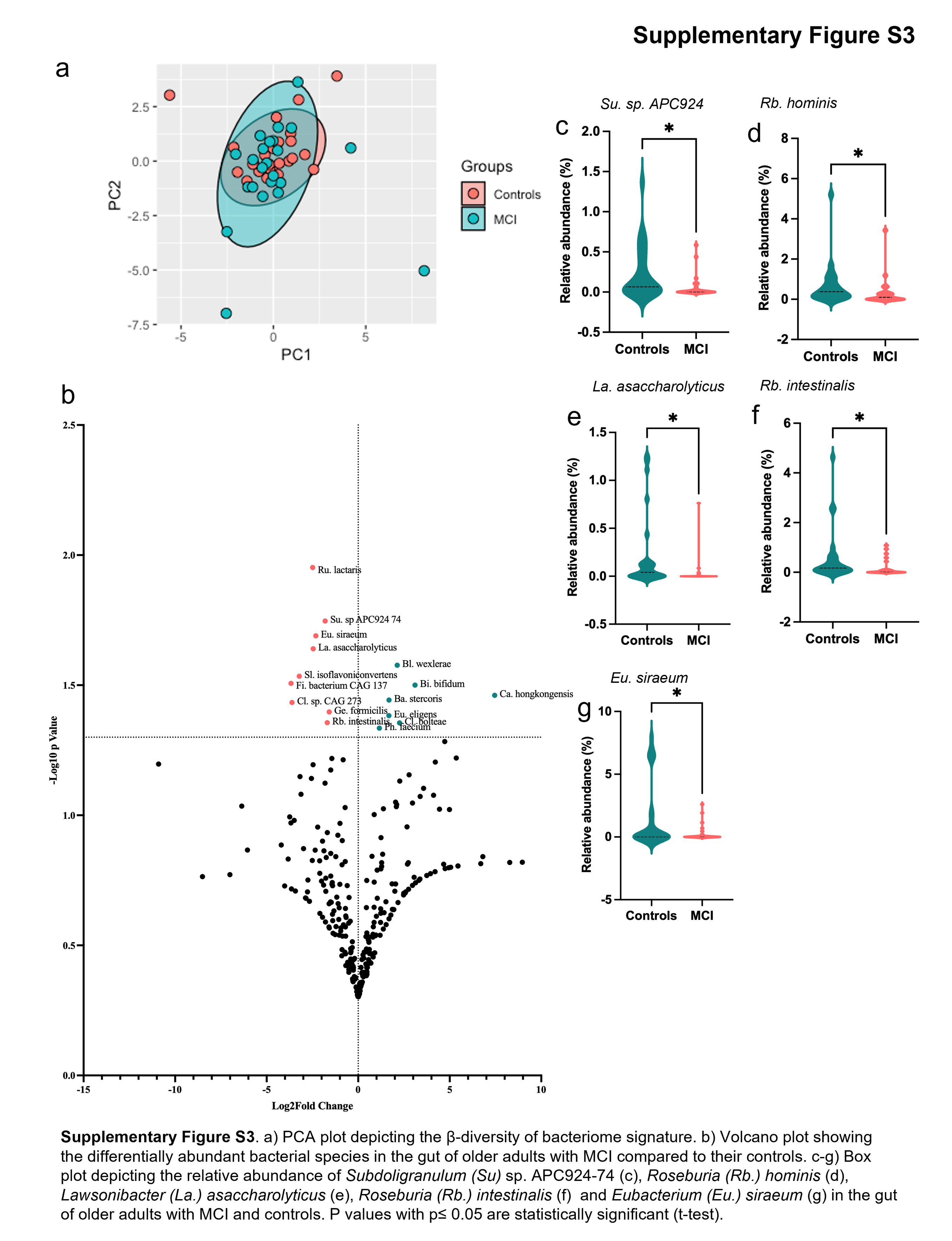

Supplement: Supplementary file 9 — a) PCA plot depicting the β-diversity of bacteriome signature. b) Volcano plot showing the differentially abundant bacterial species in the gut of older adults with MCI compared with the controls. c-g) Box plot depicting the relative abundance of Subdoligranulum (Su) sp. APC924-74 (c), Roseburia (Rb.) hominis (d), Lawsonibacter (La.) asaccharolyticus (e), Roseburia (Rb.) intestinalis (f) and Eubacterium (Eu.) siraeum (g) in the gut of older adults with MCI and controls. P values ≤ 0.05 are statistically significant (t-test). (PNG 1001 kb) [file 11357_2023_799_Fig8_ESM.png]

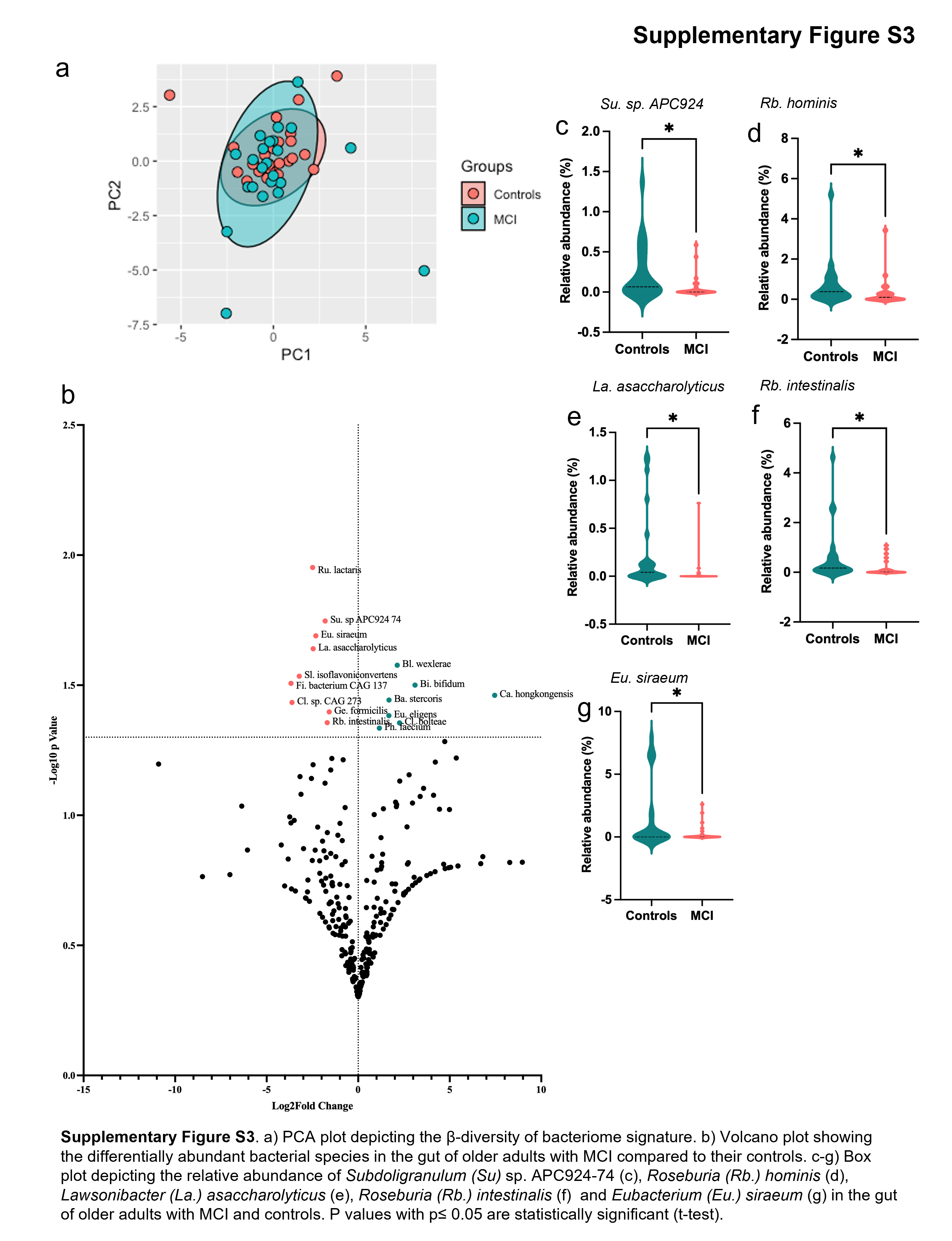

Supplement: Supplementary file 10 — High resolution image (TIF 1958 kb) [file 11357_2023_799_MOESM7_ESM.tif]

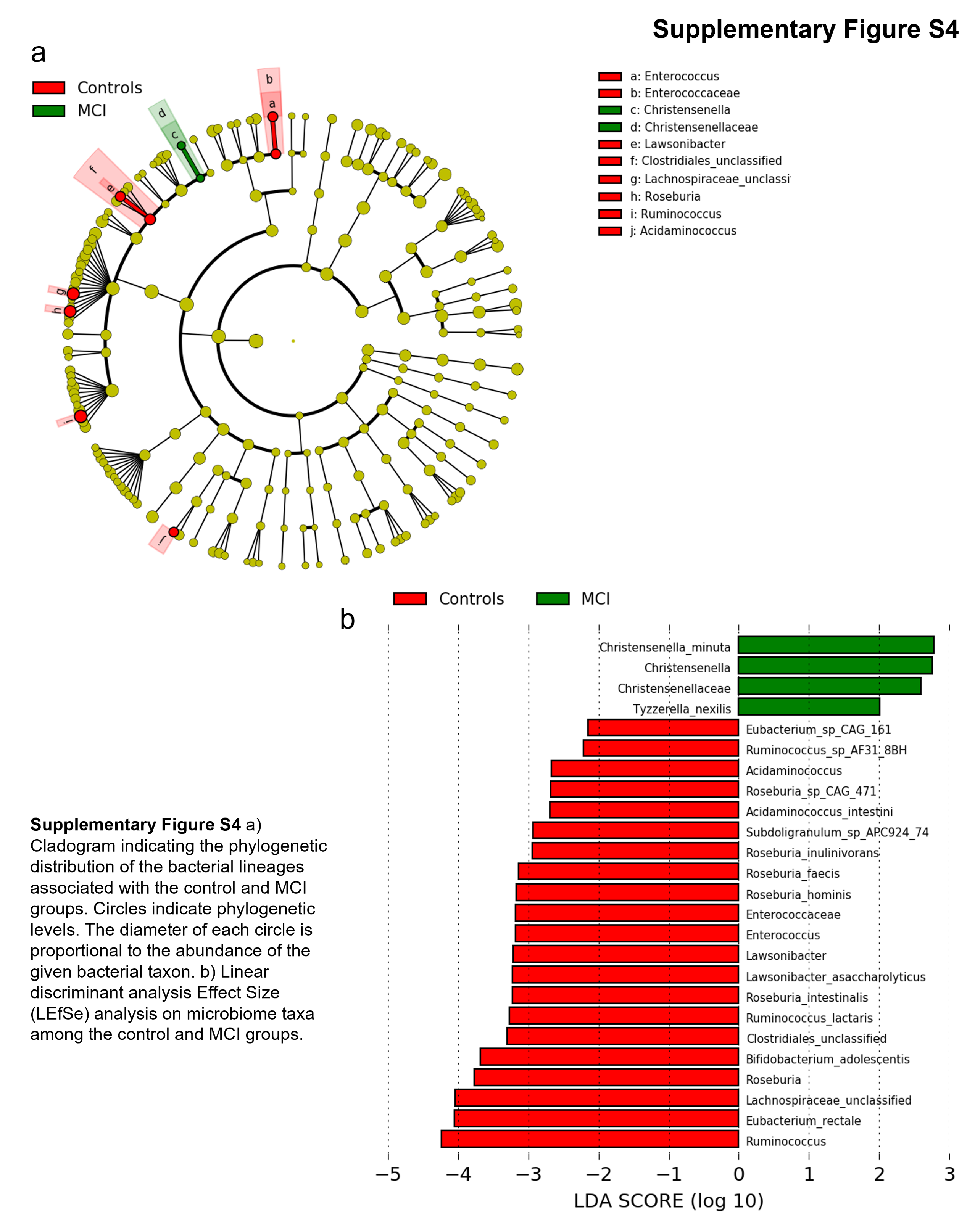

Supplement: Supplementary file 11 — a) Cladogram indicating the phylogenetic distribution of the bacterial lineages associated with the control and MCI groups. Circles indicate phylogenetic levels. The diameter of each circle is proportional to the abundance of the given bacterial taxon. b) Linear discriminant analysis Effect Size (LEfSe) analysis on microbiome taxa among the control and MCI groups. (PNG 1544 kb) [file 11357_2023_799_Fig9_ESM.png]

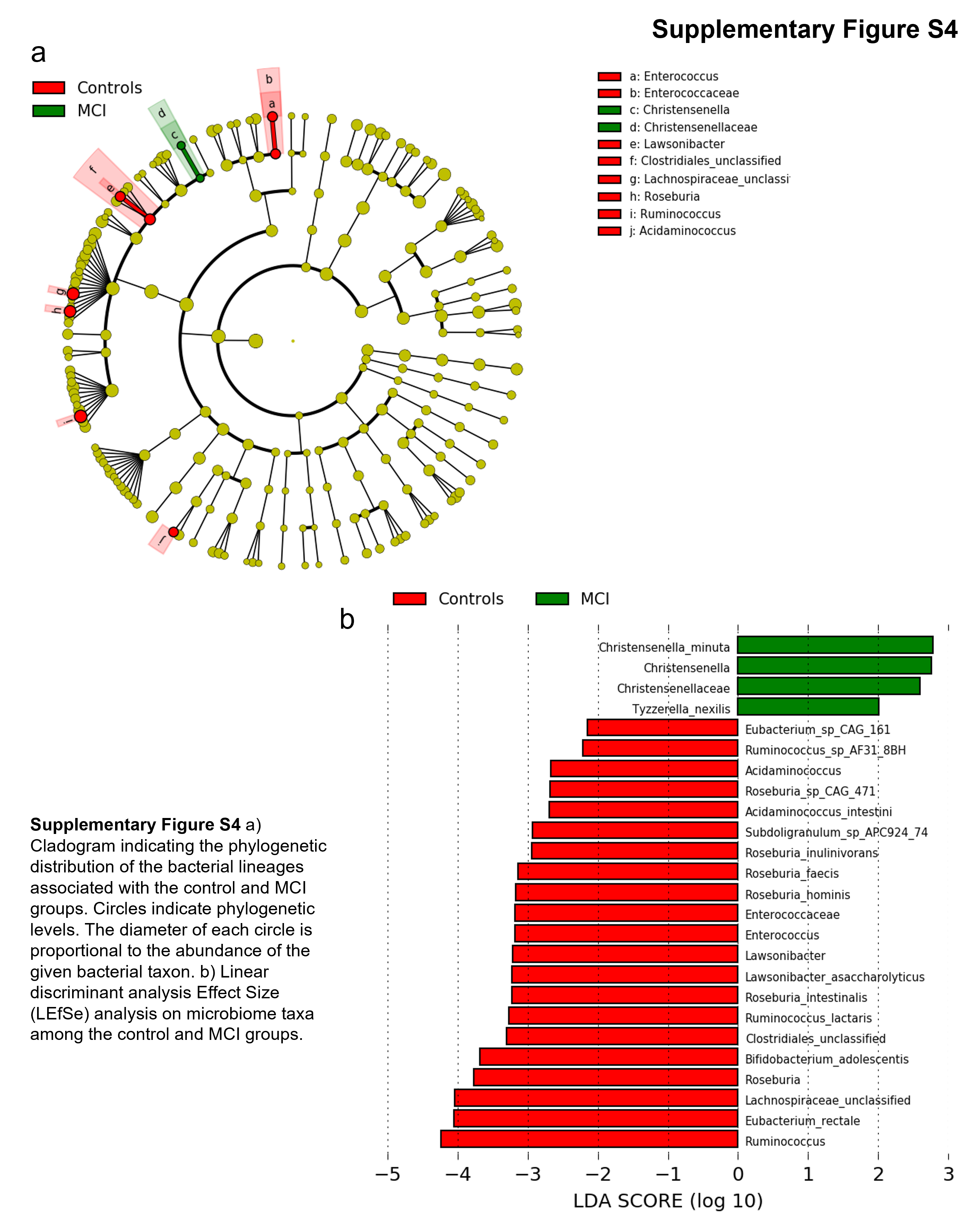

Supplement: Supplementary file 12 — High resolution image (TIF 2492 kb) [file 11357_2023_799_MOESM8_ESM.tif]

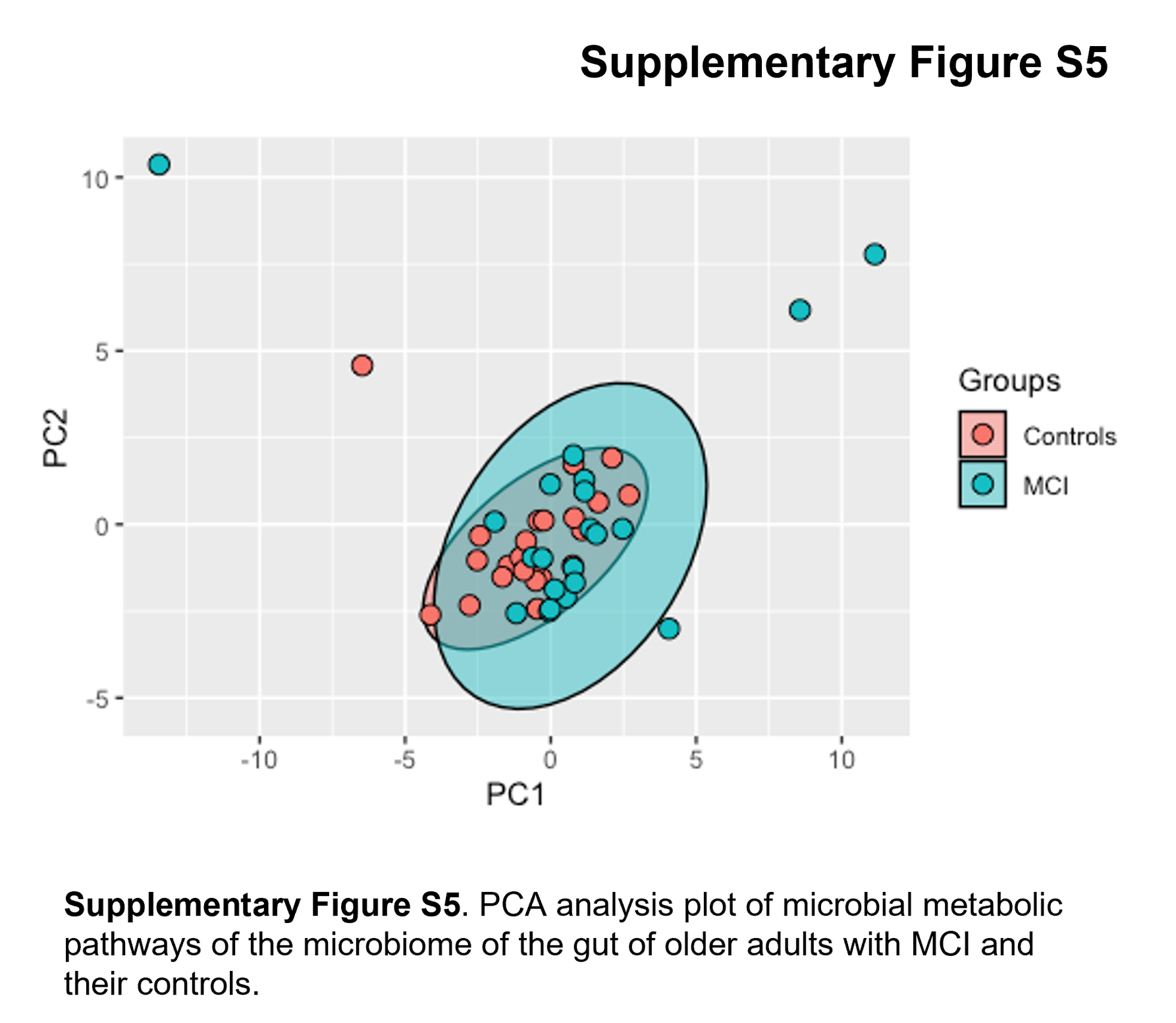

Supplement: Supplementary file 13 — PCA analysis plot of microbial metabolic pathways of the gut microbiome of older adults with MCI and controls. (PNG 323 kb) [file 11357_2023_799_Fig10_ESM.png]

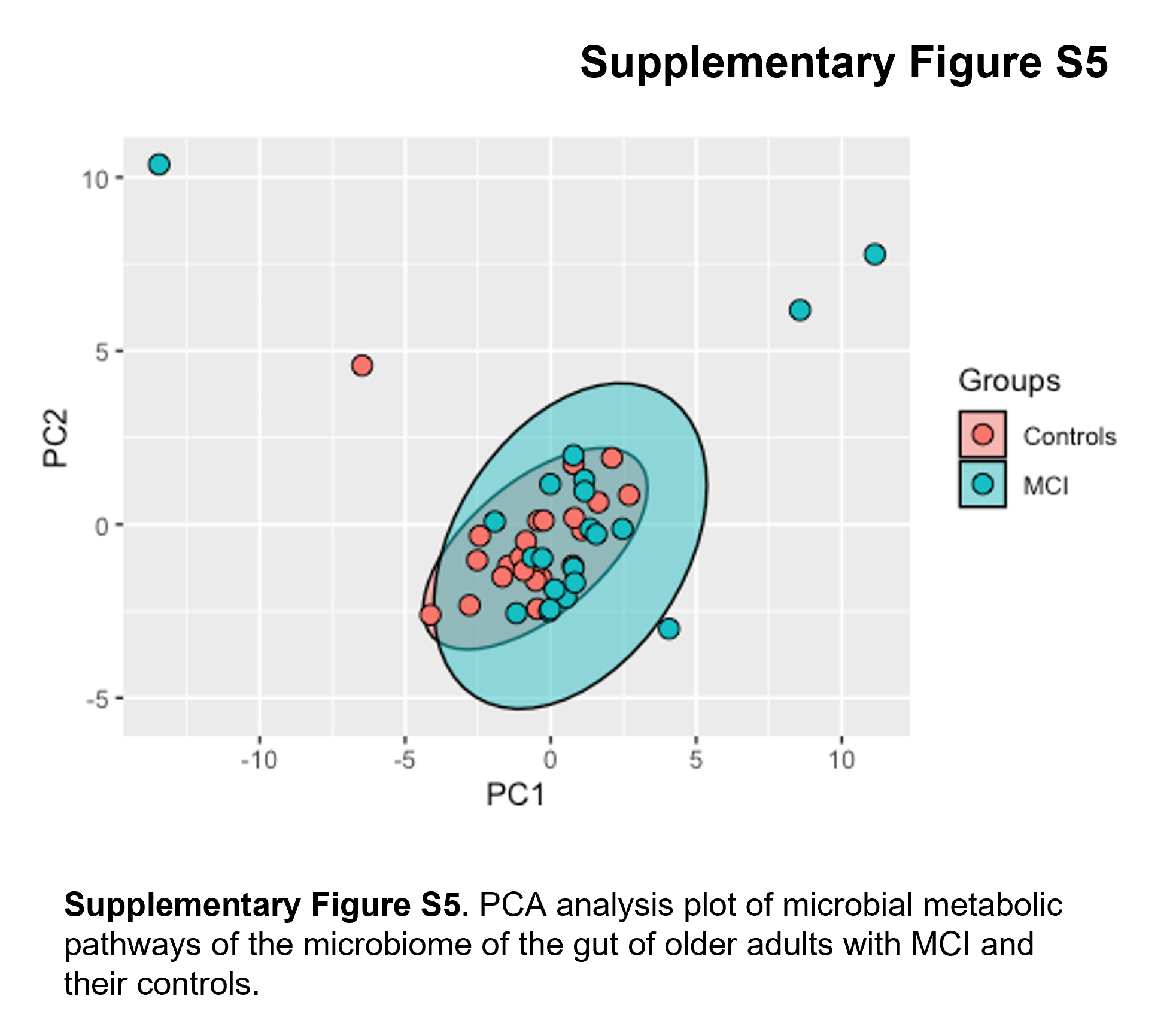

Supplement: Supplementary file 14 — High resolution image (TIF 874 kb) [file 11357_2023_799_MOESM9_ESM.tif]

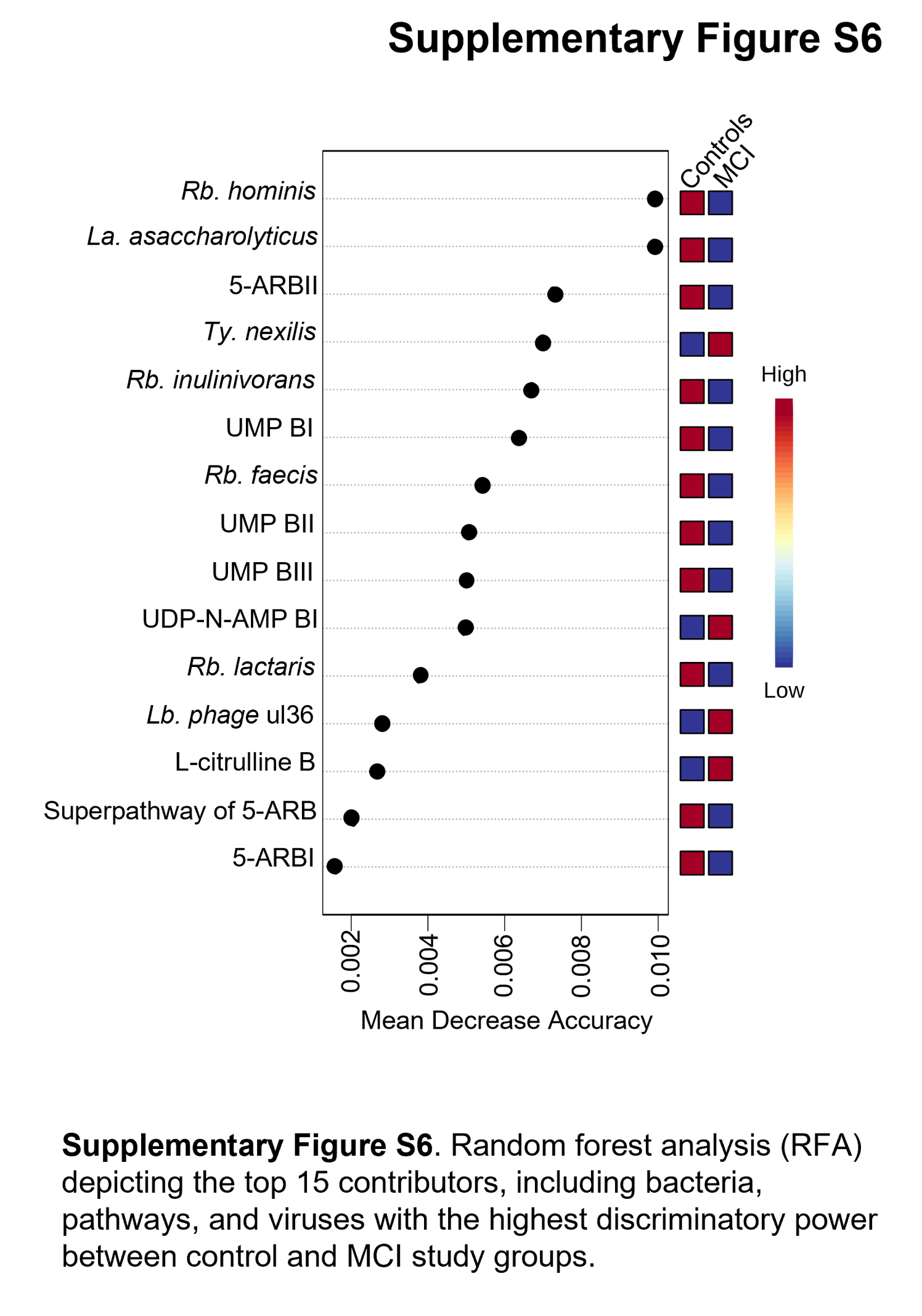

Supplement: Supplementary file 15 — Random forest analysis (RFA) depicting the top 15 contributors, including bacteria, pathways, and viruses with the highest discriminatory power between the control and MCI groups. (PNG 348 kb) [file 11357_2023_799_Fig11_ESM.png]

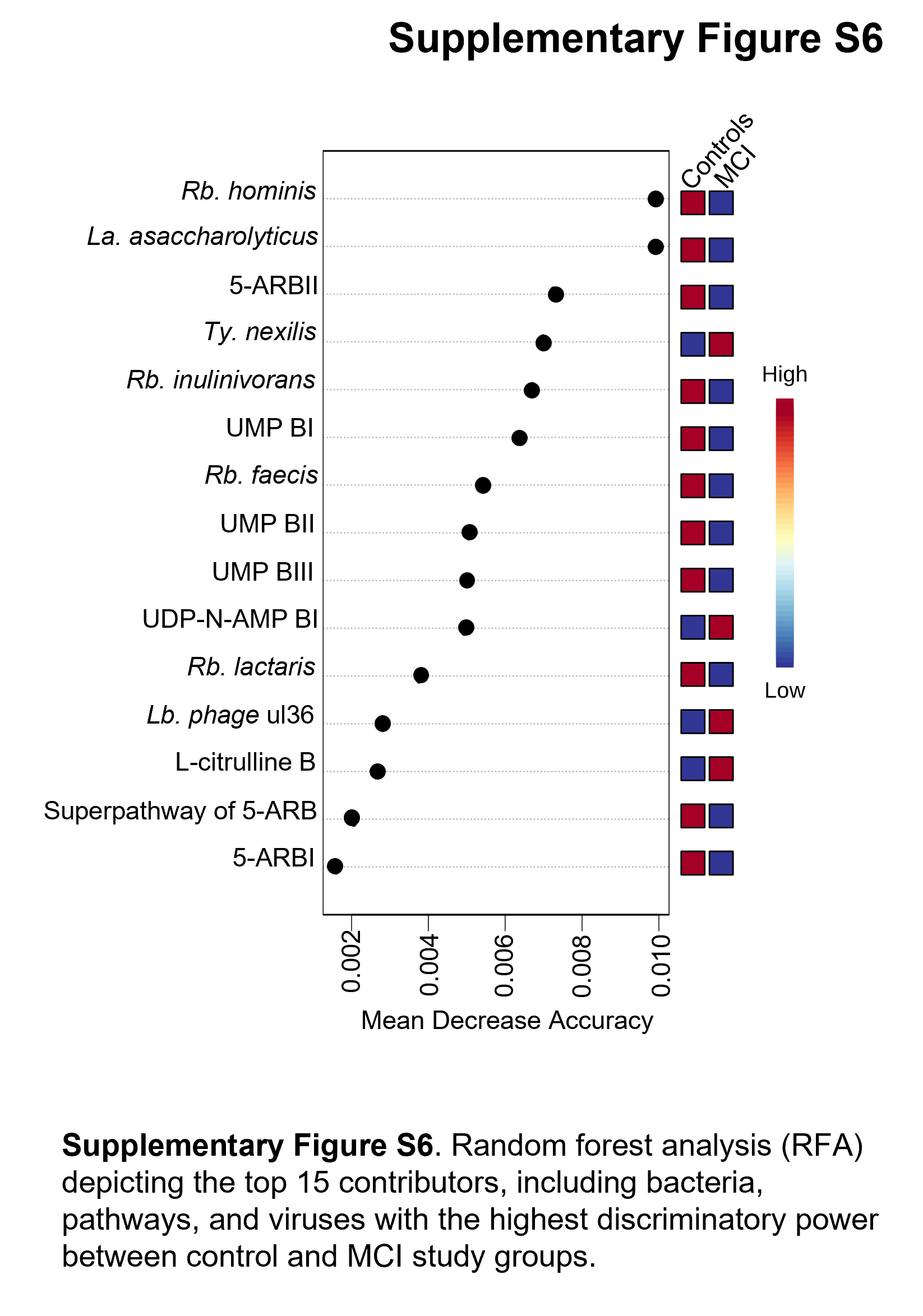

Supplement: Supplementary file 16 — High resolution image (TIF 605 kb) [file 11357_2023_799_MOESM10_ESM.tif]
